# Developing a predictive model for blood-brain-barrier permeability to explore relevance of *in vitro* neurotoxicity data for *in vivo* risk assessment

**DOI:** 10.3389/ftox.2025.1535112

**Published:** 2025-04-17

**Authors:** Siena E. Illa, Yumei Feng Earley, Li Li, Dingsheng Li

**Affiliations:** ^1^ School of Public Health, University of Nevada, Reno, NV, United States; ^2^ Department of Medicine, University of Rochester Medical Center, Rochester, NY, United States; ^3^ Department of Pharmacology and Physiology, University of Rochester Medical Center, Rochester, NY, United States

**Keywords:** blood-brain-barrier, permeability, neurotoxicity, *in vitro*, *in vivo*, toxicokinetic

## Abstract

**Introduction:**

Despite recent rapid advancements in *in vitro* toxicology, its application to whole-body health outcomes remains limited. Incorporating factors like internal exposure, such as permeability across biomembranes, could improve its relevance. Notably, there is a lack of data and predictive models for blood-brain barrier (BBB) permeability, a proxy for the exposure of target organs to neurotoxicity. We developed a predictive model for BBB permeability to investigate whether it can strengthen the correlation between *in vitro* and *in vivo* neurotoxicity data.

**Methods:**

We collected permeability data from parallel artificial membrane permeability assays for brain membranes (PAMPA-BBB) for 106 compounds with varied physicochemical properties. This was utilized to develop an empirical model to expand the potential coverage of chemicals. A list of 23 chemicals with available *in vivo* and *in vitro* neurotoxicity data from EPA IRIS and ToxCast was curated to analyze the correlation in toxicity rankings with the Spearman correlation coefficient, with and without the consideration of permeability from our predictive model.

**Results:**

The PAMPA-BBB predictive model showed promising results, with an R2 of 0.71 (measured vs predicted permeabilities). Considering permeability did not improve the correlation between *in vitro* and *in vivo* neurotoxicity (0.01 vs -0.11).

**Discussion:**

This weak correlation may stem from model uncertainty and the exclusion of other toxicokinetic processes, along with interspecies toxicodynamics differences. Our results indicate more detailed information on how neurotoxic substances behave inside the body is essential to better utilize the *in vitro* neurotoxicity data for predicting *in vivo* toxicity and assessing the risk to the central nervous system.

## 1 Introduction

The focus of development in chemical risk assessment has recently shifted from traditional *in vivo* testing towards *in vitro* approaches (Yoon et al., 2012), collectively referred to as new approach methodologies (NAMs) (U.S. EPA, 2020). The use of *in vitro* assays is promoted for several reasons, including (1) being more ethical; (2) facilitates easier and more affordable replication (Daniel, 2018; Zhao, 2023); and (3) allows for species-specific cell investigations (Nesslany, 2017).

However, applying *in vitro* testing results to *in vivo* risk assessment contexts presents challenges (Zhang et al., 2018). For example, while *in vitro* assays can efficiently assess the direct toxic effects of chemicals on target cells, they do not accurately reflect how these chemicals behave in the integrated systems of a living organism (Yoon et al., 2012). *In vitro* assays may not account for the metabolic processes that occur *in vivo*, which can alter the toxicity profile of a chemical (Wilk-Zasadna et al., 2015). To overcome these challenges, research has integrated physiologically based pharmacokinetic (PBPK) models to translate *in vitro* findings into predictors of *in vivo* behavior (Bessems et al., 2014; Yoon et al., 2012). Converting *in vitro* concentration-response data into *in vivo* dose-response relationship with reverse dosimetry techniques has been successful in predicting developmental (Louisse et al., 2010; Strikwold et al., 2013), kidney (Abdullah et al., 2016), and liver toxicity (Chen et al., 2018).

*In vitro* to *in vivo* extrapolation (IVIVE) studies focusing on neurotoxicity are rare. In a high throughput toxicokinetic (HTTK) evaluation of the predictive accuracy of *in vitro* bioactivity for *in vivo* adverse effects by Friedman et al. (2020) neurotoxic studies were included, among other toxicity endpoints. However, the current version of HTTK does not specialize in assessing the potential correlation between *in vivo* and *in vitro* neurotoxicity data but provides a more generalized comparison between *in vivo* and *in vitro* point-of-departures (PODs). When comparing *in vitro* and *in vivo* neurotoxic data, the interaction of the blood-brain barrier (BBB) may complicate analysis. The BBB acts as a protective barrier, restricting the access of certain chemicals to the central nervous system (CNS) (Karami-Mohajeri and Abdollahi, 2011). The ability of a substance to permeate across the BBB is crucial in determining its potential neurotoxic effects, potentially leading to more severe neurotoxic outcomes as they can interact with the CNS more directly (Mikitsh and Chacko, 2014).

Given the importance of BBB in determining neurotoxicity on the *in vivo* level, methods and data addressing permeability across the BBB are needed. The parallel artificial membrane permeability assay (PAMPA) is a high-throughput method consisting of a phospholipid barrier that mimics cellular membranes, widely used for estimating the permeability of chemicals (Sun et al., 2017). PAMPA has been adapted to model various biological membranes, including the BBB, using porcine brain lipids (PBL) (Mensch et al., 2010). Despite PAMPA’s ability to measure the effective permeability (Pe) of chemicals, little research has focused on leveraging it to assess the interaction between chemicals of environmental concern and the BBB. This may be due to the physicochemical properties of chemicals of environmental concern may differ from common PAMPA experimental settings that are optimized for pharmaceuticals of different physicochemical property space (Intasiri et al., 2024). Recently, Kato et al. (2023) released a model for BBB permeability measured by PAMPA. However, Kato’s model is a classifier, where the accuracy is assessed by its ability to classify a chemical’s permeability into “high” (Pe > 10 × 10^−6^ cm/s) and “low” (Pe ≤ 10 × 10^−6^ cm/s). Moreover, this model, like many others, largely considers pharmaceuticals. Therefore, there is a need for a specialized model to accurately assess the BBB permeability of chemicals of environmental concern.

In our previous work (Wang et al., 2022), we developed a silico mass balance model to assess the applicability of chemicals of environmental concern (CEC) to PAMPA, considering how chemical properties such as hydrophobicity and volatility influence their sensitivity to PAMPA experimental conditions. In the following paper (Intasiri et al., 2024), we tested 51 compounds, primarily CECs, under both PAMPA-GIT and PAMPA-BBB conditions. We then further evaluated the previously mentioned model incorporating BBB conditions, allowing comparison between the predicted Pe results and measured data. Given the gap of knowledge in BBB permeability for chemicals of environmental concern and how it can improve the interpretation of *in vitro* toxicity testing results, this study seeks to explore the predictive power of *in vitro* assays in terms of both permeability across the BBB and toxicity to CNS cells in the context of leveraging these NAM for *in vivo* chemical risk assessment for neurotoxicity. To accomplish this overall objective, we first developed a PAMPA-BBB model based on experimental results more suitable to predict the permeability of chemicals of environmental concern. Then, we examined the relationship between *in vitro* and *in vivo* neurotoxicity testing results with and without the consideration of BBB permeability to explore whether we can enhance the utility of *in vitro* neurotoxicity testing in the context of *in vivo* chemical risk assessment after considering BBB permeability.

## 2 Materials and methods

### 2.1 PAMPA-BBB model development

Previously, we produced PAMPA-BBB experimental results (effective permeability, Pe) for 30 chemicals mostly comprised of environmental pollutants (Intasiri et al., 2024). In brief, a clear PAMPA 96-well filter plates (hydrophobic PVDF membrane, 0.45 µm, non-sterile) was used. The filter of each PAMPA-BBB well was coated with 4 µL membrane consisting of 2% (w/v) PBL in dodecane. All PAMPA assays were performed in PBS (pH 7.4) containing 1% DMSO as a transport solvent unless stated otherwise. Experiments were given a 30-minte permeation period at room temperature. For more details, refer to (Intasiri et al., 2024). Due to non-quantitative results, only 30 out of 51 chemicals were viable to use in this study. To strengthen the sample size of data to build the model, we additionally extracted 106 Pe values from Di et al. (2003), Müller et al. (2015), and Vucicevic et al. (2015). This was done by prioritizing published results with similar or identical experimental conditions, including incubation time (18 h) and pH (7.4). This is because the validity of PAMPA results is reliant on setting up appropriate experimental conditions that may vary based on the physicochemical properties of the investigated chemicals. Chemicals with lower solubility and stability, such as chemicals of environmental concern, require longer incubation times to achieve accurate measurements (Sun et al., 2017; Wang et al., 2022). After removing 27 duplicates, as well as three measurements of Pe = 0 originating from Di et al. (2003), we ended up with a final sample size of 106. For the complete information on the Pe results, experimental conditions, and the key physicochemical properties of the chemicals used in this study, please refer to Supplementary Tables S1, S2.

In this study, we utilized the PaDEL-Descriptor software to compute molecular descriptors and fingerprints for our target chemicals. Developed in Java, PaDEL-Descriptor, short for “Public Domain Chemical Descriptor Library,” plays a vital role in computational chemistry and drug discovery (Yap, 2011). Molecular descriptors, grouped by 1D and 2D characteristics (n = 1,444), were generated for the chemicals under investigation. To initiate our analysis, we gathered SMILES representations of 106 chemicals from the EPA Comptox dashboard. These representations were then processed through PaDEL-Descriptor to extract pertinent descriptors, laying the groundwork for subsequent model development.

R was used for model creation. Specifically, the package “MASS” was downloaded to conduct stepwise linear regression using the stepAIC function. This allows an automatic selection of molecular descriptors that are the most significantly correlated to log Pe from the entire set. For model evaluation, we compared the measured Pe values to the predicted values, calculating the model’s R^2^ as the measurement for its performance. In addition, we calculated the model’s balanced accuracy (BACC), as used previously by Kato et al. (2023) to compare our model’s accuracy to Kato et al. (2023), which is the only other PAMPA-BBB predictive model available (Kato et al., 2023). Specifically, chemicals were first grouped into fast (Pe > 10 × 10^−6^ cm/s) and slow (Pe ≤ 10 × 10^−6^ cm/s) permeability, BACC was calculated by averaging the sensitivity, the probability of true positive results, and specificity, the probability of the true negative results. This tests the model’s ability to accurately place a chemical in the fast or slow permeability group, compared to the original measured value placement.

$$sensitivity=\frac{TP}{TP+FN}$$

$$specificity=\frac{TN}{TN+FP}$$

$$Balanced\;Accuracy\;\left(BACC\right)=\frac{sensitivity+specificity}{2}$$

TP = true positive, FP = false positive, TN = true negative, FN = false negatives.

#### 2.1.1 *In vivo* and *in vitro* toxicity data

*In vivo* toxicity data was obtained from the Integrated Risk Information System (IRIS), a database created and maintained by the United States Environmental Protection Agency (U.S. EPA, 2024a), which documents the hazardous effects on biological systems. We collected all chemicals with documented effects tagged for the nervous system (e.g., those inducing brain ChE inhibition, neurobehavioral effects, and tremors) via ingestion exposure. Notably, Point of Departure (POD) values, i.e., the lowest doses at which a significant biological effect were directly observed in animal tests, were prioritized over Reference Dose (RfD) to avoid the influence from uncertainty factors used to derive the RfD values from POD values (U.S. EPA, 2002) In total, data for 54 chemicals were initially retrieved from the IRIS database.

*In vitro* neurotoxic bioactivity levels for chemicals were determined using assay-specific data downloaded from the US EPA’s CompTox Chemistry (U.S. EPA, 2023) following a methodology similar to Li et al. (2020). ToxCast records cell line responses to chemicals examined by bioassays, documenting AC50 values (µM), which represent the concentration at which half of the maximal activity is achieved, for each assay. We screened 54 chemicals with POD for neurotoxicity from IRIS for assays indicating adverse effects on the nervous system. AC50 values for each chemical were excluded if they were higher than the lower bound estimates, which are based on assay endpoints in “invitrodb” including multiple cell lines and technologies and represent an estimated concentration for general cytotoxicity across assays (U.S. EPA, 2024b). For chemicals where no lower bound estimates were available, the cytotoxicity median was used as a proxy. Background and control assays were also excluded from the retrieved data. Geometric means of the remaining AC50 values were then used to represent *in vitro* neurotoxicity. A geometric mean was computed to determine the cytotoxicity center by aggregating all AC50 (or lower) values from active cytotoxicity assays. Neurotoxic assays specifically sought for analysis are described in Supplementary Tables S4, S5.

Of the 54 chemicals containing IRIS *in vivo* data, 29 had documented *in vitro* assay data available. Among these, four chemicals had only one qualifying assay for analysis. While 42 chemicals considering inhalation exposure were available in IRIS specific to neurotoxicity, only one chemical without oral exposure POD from IRIS was present in ToxCast. Additionally, since chemicals inhaled may be able to bypass the BBB via the olfactory bulb route (Jeong et al., 2023), we excluded all inhalation exposure IRIS results for further analysis in this study.

### 2.2 Examining *in vitro* and *in vivo* neurotoxicity data relationship

Before examining the correlation between *in vitro* and *in vivo* neurotoxicity data, a final step of curation was carried out to exclude chemicals with a molecular weight greater than 400 g/mol. This is because larger molecules were found to face additional challenges posed by the tight cell junctions when crossing the BBB (Mikitsh and Chacko, 2014). This paracellular transport process is not captured in any way by the PAMPA. Hence, we focused on chemicals that are smaller and with both *in vivo* and *in vitro* neurotoxicity data. This left us with a total of 23 chemicals.

We ranked these 23 chemicals respective to their *in vivo* toxicity–POD from IRIS; *in vitro* toxicity–geometric mean of curated AC50 values; and BBB permeability–predicted PAMPA BBB Pe from our model as none of the 23 chemicals have measured Pe values. For both *in vivo* and *in vitro* toxicity, the lower the toxicity value, the higher the ranking. For BBB permeability, a higher Pe is indicative of a higher ranking. We then composed a permeability-adjusted *in vitro* toxicity ranking by adding the rankings of *in vitro* toxicity and BBB permeability for each chemical and ranked the resulting products from lowest to highest. This is to represent the idea of chemicals’ ability to cause harm to the nervous system after they penetrate the BBB. Hence a lower permeability can lower the expected *in vivo* toxicity inferred from *in vitro* toxicity and vice versa.

Finally, we calculated the Spearman correlation coefficient for *in vivo* toxicity ranking versus *in vitro* toxicity ranking, and for *in vivo* toxicity ranking versus permeability adjusted *in vitro* toxicity ranking to examine if considering BBB permeability can enhance the correlation between *in vitro* data with *in vivo* data for neurotoxicity. A correlation between the rankings is considered more appropriate compared to examining the correlations between the absolute values, as rankings can better accommodate the impact of uncertainties within the toxicity and permeability data.

## 3 Results

### 3.1 Overview of *in vitro* neurotoxicity data used

The number of *in vitro* neurotoxic assays varied substantially, averaging 12.9 (±7.01) assays per chemical. Among the chemicals of interest, three (i.e., methamidophos, baygon, chlorobenzilate) only had one neurotoxic assay recorded. In contrast, hexachlorophene had the highest number of available assays in the ToxCast database, totaling 51. The geometric mean derived from the assays AC50 values ranged from 0.26 μM (endrin) to 53.69 μM (merphos oxide), with the majority of chemicals (79%) below 20 μM (Table 1). A total of 72 different types of assay types were considered, with an average of 2.71 (+-2.65) chemicals per assay, ranging from 1 to 11 chemicals per assay. Notably, only 54% of assays included 3 or more chemicals, indicating low assay overlap between chemicals. For detailed information on the *in vitro* neurotoxicity data used and a description of the assays, please refer to Supplementary Tables S4, S5.

**TABLE 1:** Summary of experimentally determined *in vivo* neurotoxicity endpoint, *in vitro* neurotoxicity bioassay values, and permeability predicted by our developed PAMPA BBB^a^ model.

Chemical	CASRN^b^	IRIS POD^c^ (μM/day)	ToxCast AC50^d^ geometric mean (μM)	Predicted Pe^e^ 10^−6^ (cm/s)
Aldicarb	116-06-3	0.01	2.70	0.45
O-Ethyl O-(4-nitrophenyl) phenylphosphonothioate	2104-64-5	0.01	24.96	2.34
Endrin	72-20-8	0.025	0.26	17.6
Fenamiphos	22224-92-6	0.025	19.57	4.39
Methyl parathion	298-00-0	0.025	17.75	1.09
Disulfoton	298-04-4	0.04	5.16	7.26
Dichlorvos	62-73-7	0.05	4.78	2.49
Dimethoate	60-51-5	0.05	39.53	0.72
Ethion	563-12-2	0.05	22.17	4.91
Methamidophos	10265-92-6	0.05	15^f^	8.15
Merphos oxide	78-48-8	0.1	53.69	6.5
Acephate	30560-19-1	0.12	0.43	0.15
2,4-Dinitrotoluene	121-14-2	0.2	9.72	0.31
Naled	300-76-5	0.2	10.23	2.24
Malathion	121-75-5	0.23	13.09	0.66
Pirimiphos-methyl	29232-93-7	0.25	48.14	4.2
Baygon	114-26-1	0.36	5.09^f^	2.18
Carbofuran	1563-66-2	0.5	7.50	2.14
Phosmet	732-11-6	2	32.09	1.39
Danitol	39515-41-8	2.5	2.77	15.1
Benzidine	92-87-5	2.7	19.06	12.4
Chlorobenzilate	510-15-6	5	14.33^f^	11.0
Mepiquat chloride	24307-26-4	25	5.58	2.21

^a^ Parallel Artificial Membrane Assay- Blood Brain Barrier.

^b^ Chemical Abstracts Service Registry Number.

^c^ Integrated Risk Information System Point of Departure.

^d^ Concentration at which half of the maximal activity is achieved.

^e^ Effective permeability.

^f^ Only one value available for neurotoxicity bioassay.

### 3.2 PAMPA-BBB model evaluation

The seven most significant (p-value <0.001) molecular descriptors selected by the stepwise linear regression model included topological polar surface area (TopoPSA), smallest absolute eigenvalue of Burden modified matrix (with a path length of 5) weighted by relative first ionization potential (SpMin5_Bhi), Geary autocorrelation (considering atoms that are directly bonded) weighted by mass (GATS1m), count of E-State descriptors of strength for potential Hydrogen Bonds of path length 8(nHBINT8), structural information content index for neighborhood symmetry of 2-order (SIC2), Moran autocorrelation (lag 1) weighted by I-state (MaTS1s) and minimum atom-type H E-State for H on C vinyl bonded to C aromatic (minHAvin).

$$LogBBB=-3.288971+\left(-0.014712\times TopoPSA\right)+\left(1.595245\times SpMin5\_Bhi\right)+\left(-1.568614\times GATS1m\right)+\left(-0.467135\times nHBint8\right)+\left(-2.14832\times SIC2\right)+\left(-1.519468\times MATS1s\right)+\left(1.192349\times minHAvin\right)$$

**FIGURE 1:**
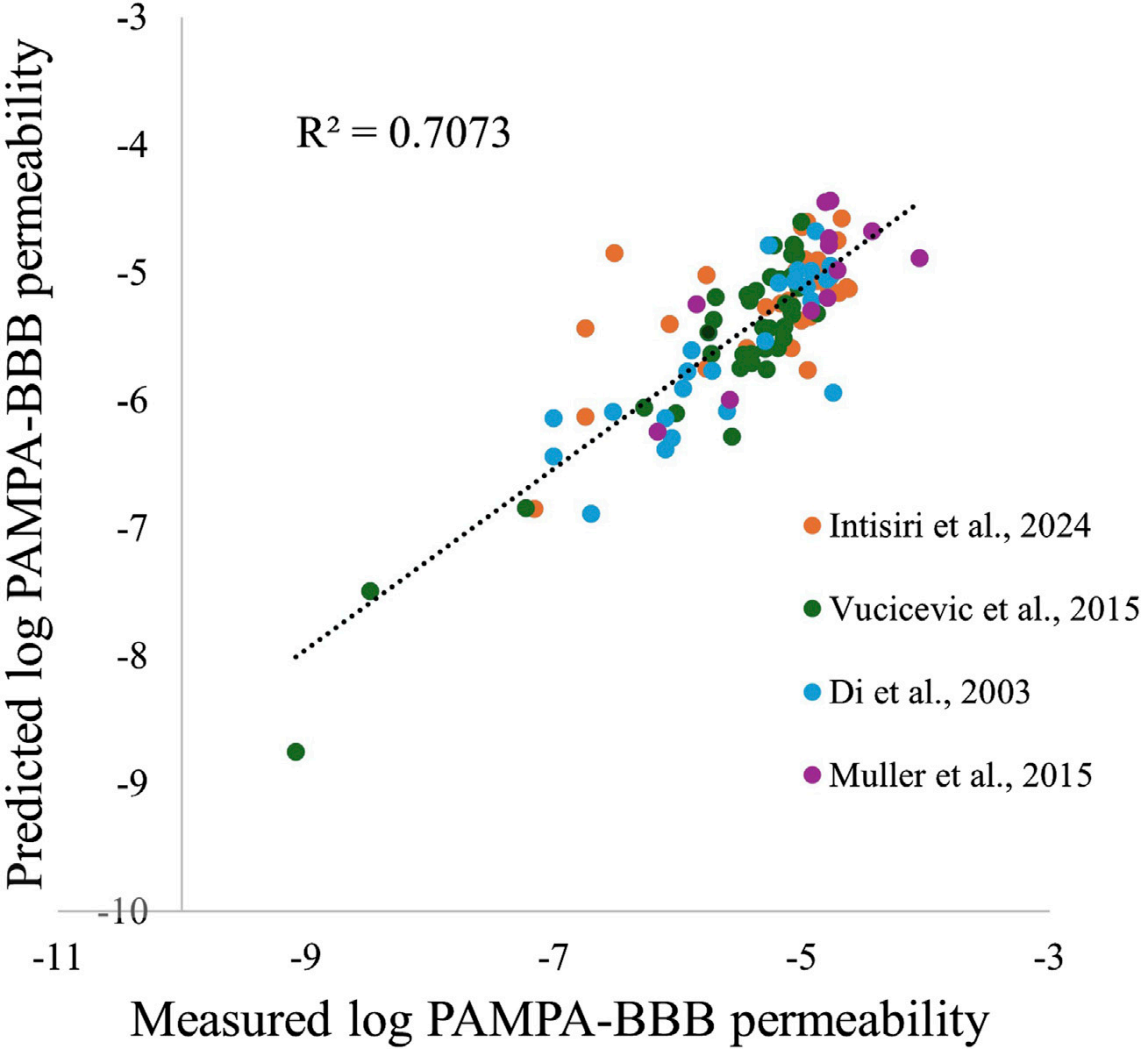
Comparison between the predicted and measured log PAMPA-BBB permeability values.

The measured Pe compared to the model-predicted Pe (n = 106), yielded an R^2^ of 0.71 and a root mean square error (RMSE) of 0.99 log units suggesting a strong ability to predict permeability based on the selected descriptors (Figure 1). The model evaluation BACC score, when implementing a cutoff of 10 × 10^−6^ cm/s, was 0.67, with a sensitivity of 0.86, and specificity of 0.48 (measured and predicted Pe values can be found in Supplementary Tables S4, S5).

### 3.3 Physicochemical property space

When comparing the physicochemical properties of the training set (n = 106) to the prediction set (n = 23), the majority of the prediction chemicals fall within the range. The log Kow values in the training set range from −4.5 to 8.35 (paraquat dichloride to alprenolol), encompassing all of the log Kow values in the prediction set (−2.82–7.54). The water solubility within the training dataset ranged from 9.12 × 10^-7^ to 2.72 mol/L (rhodamine 6G to paraquat dichloride), similar to the range of the predicted chemicals, from 6.24 × 10^-7^ to 5.2 mol/L (endrin to methamidophos). For vapor pressure, the training set ranged from 9.60 × 10^-11^ to 9.00 × 10^-2^ mmHg (Isoxicam, 2,4-dichlorophenol) with all predicted chemicals falling into the range except for mepiquat chloride (0.148 mmHg). Training set and prediction set physicochemical properties are summarized in Supplementary Tables S2, S3.

In addition, the range of molecular descriptor values for the training set covered most of the prediction set. TopoPSA training set values ranged from 3.24 to 159.29 (amitriptyline to amiloride) with all but two chemicals (mepiquat chloride and ethion) falling out of range (0 and 171.32). In contrast, SpMin5_Bhi training set values ranged from 0.16 to 1.38 (agmatine to buspirone), and encompassed all values in the prediction set (0.20–1.23). For GATS1m, training set values ranged from 0.46 to 1.28 (bromazepam to agmatine), capturing the majority of the prediction chemicals with the exception of phosmet, endrin and mepiquat chloride (0.431, 0.432 and 1.37). Training set values for nHBINT8 ranged from 0 to 4 (bromazepam to amiloride) encompassing all values in the prediction set. For SIC2, training set values ranged from 0.60 to 0.93 (paraquat dichloride to tizanidine), with merphos oxide, ethion, benzidine, and mepiquat chloride falling below range (0.53–0.59). The MaTS1s training set values ranged from −0.48 to 0.09 (amiloride to imipramine) with all prediction set chemicals falling into range (−0.40 to 0.056). Lastly, minHAvin training set values ranged from 0 to 0.75 (amiloride to vanillin) with all prediction set values falling within rage at zero.

### 3.4 *In vitro* versus *in vivo* neurotoxicity value relationship

The Spearman correlation coefficient for *in vivo* toxicity ranking versus *in vitro* toxicity ranking is 0.01 (p-value = 0.96), indicating there is virtually no correlation between the two rankings. The Spearman correlation coefficient for *in vivo* toxicity ranking versus permeability adjusted *in vitro* toxicity ranking is −0.11 (p-value = 0.61), indicating a negative correlation between the two rankings but also insignificant, and that by considering the predicted Pe, the correlation between *in vitro* and *in vivo* neurotoxicity values did not improve. Neurotoxicity rankings and Spearman correlation results are summarized in Supplementary Table S6.

## 4 Discussion

When comparing the PAMPA-BBB model predictions with the measurements, the overall RMSE of 0.99 log units was comparable to the 0.92 log units reported by Wang et al. (2022), which corresponds to 8.32. The evaluation BACC score is in line with Kato et al. (2023), whose best-performing model achieved a BACC of 0.70, with a sensitivity of 0.53 and specificity of 0.87, while their lowest-performing model had a BACC of 0.61, with a sensitivity of 0.30 and specificity of 0.92. When comparing sensitivity and specificity, our model excelled in identifying permeability values ≤ 10 × 10^−6^ (true positives), whereas Kato et al.'s model demonstrated stronger performance in identifying permeability values > 10 × 10^−6^ cm/s (true negatives). This difference may stem from the composition of our training set, where 68% of chemicals belonged to slow (Pe ≤ 10 × 10^−6^ cm/s) and 32% to fast (Pe > 10 × 10^−6^ cm/s). In contrast, Kato et al. had 30% (n = 554) of chemicals in 1% and 70% (n = 1,240) in class 0.

In this study, we developed a predictive model for PAMPA-BBB effective permeability. To our knowledge, this is one of the few models that have predictive capabilities for BBB permeability and the only one that provides quantitative estimates. While a more robust sample size would have strengthened our model, we were inherently limited by minimal PAMPA-BBB sources and adhering to select experimental conditions to ensure comparable PAMPA-BBB permeability results (Intasiri et al., 2024; Wang et al., 2022). Although this permeability does not reflect potential mechanisms, such as possible active transport for some chemicals when crossing the BBB, it can serve as an efficient high throughput estimate of the permeability of chemicals for the BBB for screening purposes. Ideally, results from a more comprehensive permeability testing protocol, such as coculturing multiple relevant types of cells in the BBB (Kulczar et al., 2017; Lubin et al., 2024) that theoretically represent a closer approximation of *in vivo* interactions between the BBB and chemicals, should be compared to the PAMPA-BBB results to assess its accuracy. However, there is a significant lack of data from cell-culture-based experiments on chemical pollutants, making such a comparison currently unfeasible. The finding that there is no correlation between *in vitro* and *in vivo* neurotoxicity data is somehow disappointing. *In vitro* toxicity assays are supposed to be a more efficient way to screen for toxicity compared to conducting traditional animal experiments so human risk assessment can be better informed when facing the ever-growing inventory of pollutants people are exposed to but lack existing toxicity data. Here, we offer several possible reasons that could make *in vitro* neurotoxicity testing results hard to relate to their *in vivo* counterparts.

First, *in vitro* studies report nominal concentrations in the apparatus that may differ from the freely dissolved concentrations that exert the actual toxic effects on the cells due to factors like binding to plastic walls, and serum proteins, as well as evaporation and degradation/biotransformation in both systems (Groothuis et al., 2015; Proença et al., 2021). Depending on the experimental setup and the properties of the chemicals, these factors can cause varying degrees of differences between the nominal and freely dissolved concentrations, with the latter considered to be a better representation of *in vitro* toxicity, yet much harder to measure (Groothuis et al., 2019; Henneberger et al., 2021). Since ToxCast reports only nominal concentrations, the ranking of *in vitro* neurotoxicity used in this study may change should the freely dissolved concentrations be known.

Second, toxicokinetics play a major role in determining how much of a chemical originally ingested (represented by *in vivo* POD) can eventually reach the target sites to induce neurotoxic effects (represented by *in vitro* AC50). This important factor has been explored earlier by researchers applying high throughput toxicokinetic models to perform *in vitro* to *in vivo* extrapolation (IVIVE) to examine how closely the two can match with each other. These results show that, in general, *in vitro* AC50 can correspond to a more protective (i.e., lower) *in vivo* POD, but great variation exists for the differences between these two, or that the correlation between these two metrics can be dependent on the *in vitro* assays and/or IVIVE methodology employed (Bianchi et al., 2024). Thus, it is possible that a chemical with less capability to reach the nervous system after being ingested may have a lower *in vivo* POD while having a higher *in vitro* AC50. Moreover, two of the 23 evaluated chemicals—mepiquat chloride and chlorobenzilate—may have *in vivo* critical effects documented as neurotoxicity, yet not originated from direct impact to the nervous system (U.S. EPA, 1988; U.S. EPA, 1989).

Third, discrepancies between the tested biological systems of *in vitro* and *in vivo* toxicity assays may give rise to different biological responses observed in the different experiments. The ToxCast values for the 23 examined chemicals are based on rats, guinea pigs, bovine, and humans, while the IRIS POD values are predominately derived from rodent experiments. It has long been known that the difference in toxicodynamics such as critical enzyme activities and/or allosteric modifiers, could result in substantially different biological responses, as the mode of action may be present in one species but absent in another (Du et al., 2018; Green, 2000; Seed et al., 2005; Suzuki et al., 1994). To this end, matching the species from which the cell lines originated and used in *in vitro* assays and the animals tested in *in vivo* experiments may mitigate the intraspecies uncertainty when comparing the toxicity values from these two types of data. However, given the already relatively small sample size of chemicals that have both types of data available, no meaningful comparison can be made with this condition as of now.

Fourth, when calculating the AC50 geometric mean, all assays related to neurotoxicity for a specific chemical were treated equally, despite inherent differences that could affect comparability. The assays vary in terms of cell type, exposure duration, and endpoints, introducing heterogeneity in the data. However, there is a limited overlap of assays across chemicals, with an average of 2.7 (±2.7) chemicals per assay. Selecting from a single cell type or endpoint would significantly reduce the number of chemicals included in the analysis, further limiting sample size. To address this, we followed the practice of previous researchers by using the geometric mean of all available assays under the lower bound cytotoxicity limit as a compromise when considering multiple different assay types. By using the lower bound cytotoxicity medians, it ensures a conservative approach by providing protective thresholds for cytotoxicity, reducing the risk of underestimating potential toxic effects. Additionally, these estimates offer consistency across studies by standardizing the inclusion criteria based on a comprehensive and widely applicable dataset.

While this study cannot address issues with nominal concentration or toxicodynamic, it attempts to add some value to the discussion of toxicokinetic in the context of comparing *in vitro* and *in vivo* neurotoxicity data. Notably, IVIVE studies conducted so far have not considered the impact of BBB, which plays a critical role in the toxicokinetic of neurotoxicity as it can limit access to the nervous system for chemicals. Indeed, to the knowledge of the authors, there is no high throughput toxicokinetic model exist yet that specifically includes any part of the nervous system as an independent compartment considering the effects of BBB. Here we use PAMPA-BBB permeability as a proxy to represent higher/lower internal exposure to the nervous system after ingestion. However, no improvement in the correlation between *in vivo* toxicity and *in vitro* toxicity was found. To some extent this is not entirely unexpected as the predicted PAMPA-BBB permeability values themselves inherit the uncertainty of the model that predicted them.

One consideration is the lack of standardization in PAMPA-BBB experimental conditions and membrane composition, leading to discrepancies in protocols across different studies. When selecting PAMPA-BBB studies for model building, emphasis was placed on ensuring uniformity in experimental conditions (i.e., pH, incubation time) across publications. However, the variations in membrane composition, such as ratio of porcine brain lipid, dodecane, and volume of transport solution varied across publications. Since membrane composition influences the correlation with *in vivo* permeability, these variations could affect the model’s predictive capability, ultimately impacting its ability to accurately reflect *in vivo* conditions. Additionally, uncertainty remains regarding whether, and to what extent, the PAMPA-BBB assay accurately represents a chemical’s mass transfer process across the BBB. PAMPA-BBB uses an artificial organic membrane to approximate the endothelial cells of the BBB. However, it is important to recognize the significant differences in biological and structural complexity between this artificial system and actual BBB endothelial cells. For instance, BBB endothelial cells contain active transport systems, such as efflux pumps and receptors, which regulate molecule movement in addition to passive diffusion that is considered alone in PAMPA-BBB. Furthermore, while the lipid composition of the PAMPA membrane is designed to mimic general lipid bilayers, it does not fully replicate the complex lipid and protein composition of BBB endothelial cell membranes. Note that permeation through the BBB is not the only toxicokinetic process that determines a chemical’s level found in the central nervous system. Subsequent partitioning between brain tissue and blood also plays a thermodynamic role in determining the brain’s capacity to retain a chemical. Notably, the high fat content of the human brain (∼60%, compared to ∼0.1% in blood) favors the partitioning and accumulation of highly lipophilic chemicals, relative to hydrophilic chemicals, in the brain. Variability in this partitioning may be a significant confounding factor contributing to the lack of correlation between *in vivo* toxicity rankings and permeability-adjusted *in vitro* toxicity. Other toxicokinetic processes between the ingestion of chemicals to contacting the BBB, such as gut absorption, partition between blood and tissue, etc., are also not considered in this study, which can also vary among chemicals based on their physicochemical properties.

To conclude, developments in understanding better the freely dissolved concentration from the nominal concentrations are preferred (Armitage et al., 2014; Fisher et al., 2019). At the same time, there is a need to expand the capability of IVIVE to include the nervous system and its unique physiology of BBB more explicitly to better infer between *in vitro* and *in vivo* dose-response toxicity relationship.

## Data Availability

The original contributions presented in the study are included in the article/Supplementary Material, further inquiries can be directed to the corresponding author.
